# Cas9 endonuclease: a molecular tool for *in vitro* cloning and CRISPR edit detection

**DOI:** 10.3389/fgeed.2025.1565297

**Published:** 2025-04-01

**Authors:** Xingliang Ma, Dhouha Kthiri, Manpartik S. Gill, Curtis J. Pozniak, Sateesh Kagale

**Affiliations:** ^1^ Aquatic and Crop Resource Development, National Research Council Canada, Saskatoon, SK, Canada; ^2^ Crop Development Centre, University of Saskatchewan, Saskatoon, SK, Canada; ^3^ Department of Biological Sciences, University of Calgary, Calgary, AB, Canada

**Keywords:** gene editing, CRISPR, *Sp*Cas9, cloning, genotyping

## Abstract

Large genetic engineering constructs often face limitations in DNA element addition or replacement due to lack of unique endonuclease recognition sites. Traditional restriction resistance methods can identify CRISPR-induced mutants efficiently, but CRISPR target sites rarely contain suitable restriction motifs. Here, we demonstrate the use of *Sp*Cas9 combined with custom synthesised sgRNAs to linearize large plasmid constructs, enabling DNA element incorporation via seamless cloning methods. Additionally, *Sp*Cas9 and custom sgRNAs were used to digest target gene amplicons for effective genotyping of CRISPR-edited mutants, allowing us to distinguish between wild-type, heterozygous, and biallelic variants. This approach provides a straightforward, highly flexible method for modifying large plasmid constructs and screening CRISPR-induced edits.

## 1 Introduction

The precise cleavage of DNA *in vitro* is a fundamental aspect of recombinant DNA technology, serving as a critical element in the field of biotechnology. Restriction enzymes that cleave DNA at specific sites became the workhorses of molecular biology (Sambrook, 2001). However, plasmid constructs used for plant genetic engineering are generally large and there are limited unique restriction sites available except at the multiple cloning sites, thus making modification(s) challenging. Additionally, different plant species could exhibit preferences for selectable markers in genetic transformation. Substituting selectable markers on established binary vectors with numerous assembled cassettes is quite challenging. Recombination-based cloning methods, such as Gateway cloning (Reece-Hoyes and Walhout, 2018) and Cre-loxP-based cloning (Abremski and Hoess, 1984), eliminate the need for linearizing or cleaving the destination vector. However, these methods require specific sequences in both the vector and inserts, which restricts the selection of cloning sites in destination vectors. Additionally, despite advancements, many seamless cloning instances still require linearization of the destination vector, often through restriction enzyme-mediated cleavage. Consequently, there is a need to explore alternative and flexible approaches for digesting/linearizing vectors at desired locations and ligating DNA fragments, without relying on sticky end compatibility.

With the growing popularity of CRISPR/Cas9 (Clustered Regularly Interspaced Short Palindromic Repeats/CRISPR-associated protein 9)-based gene editing, the need for an efficient and universal method for preliminary screening of induced edits from regenerated transgenic plants and their progenies has become essential prior to further confirmation through sequencing techniques. Restriction enzyme digestion-suppressed PCR (RE-PCR) combined with agarose electrophoresis techniques can be employed for the initial screening of gene edits (Ma et al., 2015). However, the restriction resistance method, while robust, imposes significant limitations on the selection of suitable targets. The T7 endonuclease 1 (T7E1) (Kim et al., 2009) and SURVEYOR assay (Cong et al., 2013) have also been utilized to detect mutations by cleaving the hybrid molecules formed during PCR amplification of the target site, yet the resolution of the T7E1 method on agarose gel is compromised due to the low proportion of hybrid DNA molecules within the total amplicon pool and the non-specific endonuclease activity of T7E1 in cases of overreaction.

*Sp*Cas9 comprises two nuclease domains, namely the HNH domain and the RuvC-like domain (Jinek et al., 2012). The HNH domain is responsible for cleaving the complementary DNA strand to the single guide RNA (sgRNA), while the RuvC-like domain cleaves the non-complementary strand. The mature sgRNA directs *Sp*Cas9 protein to the target DNA sequence, and the ribonucleoprotein (RNP) complex identifies the target site for cleavage based on the 20-nucleotide guide sequence in the sgRNA. The DNA cleaving capability of *Sp*Cas9 can serve as a valuable tool for manipulating plasmid constructs and screening gene edits, in addition to its application in precise genome editing. In this study, we demonstrate the effectiveness of *Sp*Cas9 (*Streptococcus pyogenes* Cas9) endonuclease and it’s *in vitro* DNA cleaving ability in manipulating plasmid constructs and screening mutations in gene-edited plants.

## 2 Materials and methods

### 2.1 *In vitro* sgRNA preparation

The sgRNAs were synthesized *in vitro* using either the EnGen sgRNA Synthesis Kit (NEB, catalog no. E3322S) or the HiScribe T7 Quick High Yield RNA Synthesis Kit (NEB, catalog no. E2050S), according to the manufacturer’s protocols. All primers and oligonucleotides were synthesized by IDT and prepared at a working concentration of 5 µM. For transcription with the EnGen sgRNA Synthesis Kit, the oligonucleotides were designed to incorporate the T7 RNA polymerase promoter, the target sequence (Supplementary Table S1), and an anchor sequence containing the sgRNA scaffold. These oligonucleotides were directly used in the transcription reaction following the supplier’s instructions. For transcription using the T7 Quick High Yield RNA Synthesis Kit, an additional sgRNA-R primer (2-75; Supplementary Table S1) was used to amplify the double-stranded DNA template for sgRNA production by T7 RNA polymerase. The amplification was performed with 2x Q5 Master Mix (NEB, M0494S), using the pYLsgRNA-TaU3 vector (adapted from Ma et al., 2015) as the template. The purified amplicons were subsequently used as templates for sgRNA transcription in accordance with the kit’s protocol. The resulting sgRNAs were purified using the RNA Cleanup Kit (NEB, T2040), quantified with a NanoDrop spectrophotometer, and stored at −80°C. The typical yield of purified sgRNA ranged from 10 μg to 60 µg.

### 2.2 *In vitro* plasmid cloning by *Sp*Cas9 and custom prepared sgRNA

The *Sp*Cas9 protein was purchased from NEB (Cat. No. M0386T). The eGFP coding sequence was amplified from the pGFPGUSplus vector using the 3-61 and 3-62 primer pair (Table 1) with KOD FX polymerase (MilliporeSigma, 719753). The pGFPGUSplus vector was kindly provided by Claudia Vickers (Addgene plasmid #64401; http://n2t.net/addgene:64401; RRID: Addgene_64401). The GRF4-GIF1 cassette was amplified from the JD633 vector (Debernardi et al., 2020; Addgene plasmid #160393) as the template.

**TABLE 1:** Oligoes used in this study.

ID	Sequence	Purpose
4–2	TTC​TAA​TAC​GAC​TCA​CTA​TAG​GGC​GCA​GAA​GCT​GGA​GCA​GCG​TTT​TAG​AGC​TAG​A	Template for sgRNA targeting RhtB1 amplicon
3–60	TTC​TAA​TAC​GAC​TCA​CTA​TAG​GAG​AAA​CTC​GAG​TCA​AAT​CTG​TTT​TAG​AGC​TAG​A	Template primer for sgRNA targeting BAR gene in binary vector
2–82	TTC​TAA​TAC​GAC​TCA​CTA​TAG​CCG​AAT​TAA​TTC​GGG​GGA​TCG​TTT​TAG​AGC​TAG​A	Template primer for sgRNA targeting binary vector pUbi-B
2–83	TTC​TAA​TAC​GAC​TCA​CTA​TAG​CCA​GAT​CCC​CCG​AAT​TAA​TTG​TTT​TAG​AGC​TAG​A	Template primer for sgRNA targeting binary vector pBUN421
2–75	AGCACCGACTCGGTGCC	Reverse primer for amplifying sgRNA template
1–67	ACC​GCG​CAC​GAT​CCC​AAG​AG	Primer pair for amplifying Q gene fragment
1–68	GAG​CAG​GCG​TGA​TTA​GTT​TTA​GG	
1–60	GGC​AAG​CAA​AAG​CTT​GAG​ATA​GA	Primer pair for amplifying RhtB1a gene fragment
5–12	CGG​TGA​AGT​GGG​CGA​ACT​TG	
3–61	GTC​CTG​CCC​GTC​ACC​GAG​ATT​GCC​GCT​GCC​ATG​GTG​AGC​AAG​GGC​GAG​GAG​CTG	Primer pair for amplifying eGFP coding sequence
3–62	GGA​GAA​ACT​CGA​GTC​AAA​CTA​CTT​GTA​CAG​CTC​GTC​CAT​GCC	
3–23	AAT​CCA​GAT​CCC​CCG​AAT​TAG​TGC​AGC​GTG​ACC​CGG​TCG​T	Primer pair for amplifying GRF4-GIF1 gene cassette
3–24	TGT​ACT​GAA​TTA​ACG​CCG​AAT​GAT​CTA​GTA​ACA​TAG​ATG​ACA​C	

To insert the eGFP coding sequence, the plasmid (pBUN421 or pYLCRISPR/Cas9pUbi-B) was digested in a 50 µL reaction containing 1x NEB rBuffer 3.1, 0.5 µL of 20 µM *Sp*Cas9, 1 µg of purified sgRNA3-60 targeting the BAR gene, and 0.5 µg of plasmid substrate. Table 2 provides details on the concentration conversion for *Sp*Cas9 protein, sgRNA, and substrate DNA. The reaction was incubated at 37°C for 1 h, followed by enzyme inactivation at 65°C for 10 min. The resulting linearized product was purified and combined with the eGFP amplicon using the NEBuilder HiFi DNA Assembly Cloning Kit (NEB, E5520), then transformed into *E. coli* electrocompetent cells. Recombinant clones were screened and verified using colony PCR, restriction analysis, and Sanger sequencing. The pBUN421 plasmid was generously provided by Qi-Jun Chen (Addgene plasmid #62204; http://n2t.net/addgene:62204; RRID: Addgene_62204). A similar procedure was used for cloning the GRF4-GIF1 cassette, with the exception that sgRNA2-83 was used to digest pBUN421.

**TABLE 2:** Conversion of mass concentration to molar concentration for sgRNA, Cas9 protein, and DNA substrate.

	Mass concentration (µg/µL)	Molecular weight	Mole concentration (µM)
*Sp*Cas9	3.20	160 kDa	20
sgRNA	0.20-1.25**	About 32,327 g/mol*	6.18–38.67
DNA (0.5 kb)	0.1	Average 308006.04 g/mol***	0.324
DNA (5 kb)	0.10	Average 3079736.04 g/mol***	3.25 × 10^−2^
DNA (10 kb)	0.10	Average 6159436.04 g/mol***	1.62 × 10^−2^
DNA (15 kb)	0.10	Average 9239136.04 g/mol***	1.08 × 10^−2^
DNA (20 kb)	0.10	Average 12318836.04 g/mol***	0.81 × 10^−2^

* Depending on the target sequence composition (1 μg/μL = 1/32327 × 10^6^ μM).

** Depending on the final sgRNA, yield.

*** Refers to the NEBioCalculator software (https://nebiocalculator.neb.com/).

### 2.3 Protoplast transformation and fluorescence analysis

Fifty milliliters of *E. coli* carrying the plasmid with the eGFP reporter gene were cultured overnight at 37°C with shaking at 200 rpm. The plasmid was subsequently extracted and purified using the QIAGEN Plasmid Mini Kit (Cat. No. 12123). Protoplasts were isolated from durum wheat cultivar CDC Fortitude (Pozniak et al., 2015) following the protocol described by Luo et al. (2022) and transformed with constructs containing the eGFP reporter gene. Fluorescence analysis was conducted using a Zeiss fluorescence microscope. Transformation efficiency was determined by counting the total number of protoplasts and those displaying green fluorescence, utilizing ImageJ software (https://imagej.net/ij/).

### 2.4 *In vitro* gene edit screening

For direct treatment of genomic DNA (gDNA), samples from wild-type CDC Fortitude plants were extracted using the SDS method described by Pallotta et al. (2003). One microliter of gDNA (approximately 700 ng) was combined with 0, 0.1, 0.2, 0.3, or 0.4 µM *Sp*Cas9 enzyme and the sgRNA 4-2 complex in a 10 µL reaction prepared with 1x NEB rCutSmart Buffer. The reaction was incubated at 37°C for 1 h, after which 1 µL of the reaction mixture was used as a PCR template. PCR was performed with KOD FX polymerase (Fisher, 719753) and primer pairs 1-60 and 5-12 (Table 1). Additionally, primer pairs 1-67 and 1-68 (Table 1), targeting the Q gene, were included in the same reaction as internal controls.

For genotyping PCR amplicons from gene-edited plants, the RhtB1 gene region was amplified using KOD FX and primer pairs 1-60 and 5-12 (Table 1). The resulting PCR products were purified and quantified with a NanoDrop spectrophotometer. Between 100 and 500 ng of purified PCR amplicons were then treated with a 0.5 µM *Sp*Cas9 and sgRNA 4-2 RNP complex. After heat inactivation, 10 µL of the digestion products were analyzed on a 1% agarose gel. To prevent sgRNA from appearing as a smear on the agarose gel, RNase A was added before gel analysis.

### 2.5 Alphafold3 protein modeling, molecular graphics and analysis

The protein sequences of the BAR and BAR-eGFP gene products (Supplementary Note S1) were used as input for the AlphaFold3 server. Molecular graphics were generated and analyzed using UCSF ChimeraX software. UCSF ChimeraX, developed by the Resource for Biocomputing, Visualization, and Informatics at the University of California, San Francisco, was utilized for molecular graphics and analyses, with support from the National Institutes of Health (R01-GM129325) and the Office of Cyber Infrastructure and Computational Biology, National Institute of Allergy and Infectious Diseases.

## 3 Results and discussion

### 3.1 Modification of binary vectors using *Sp*Cas9 and custom-designed sgRNAs

**FIGURE 1:**
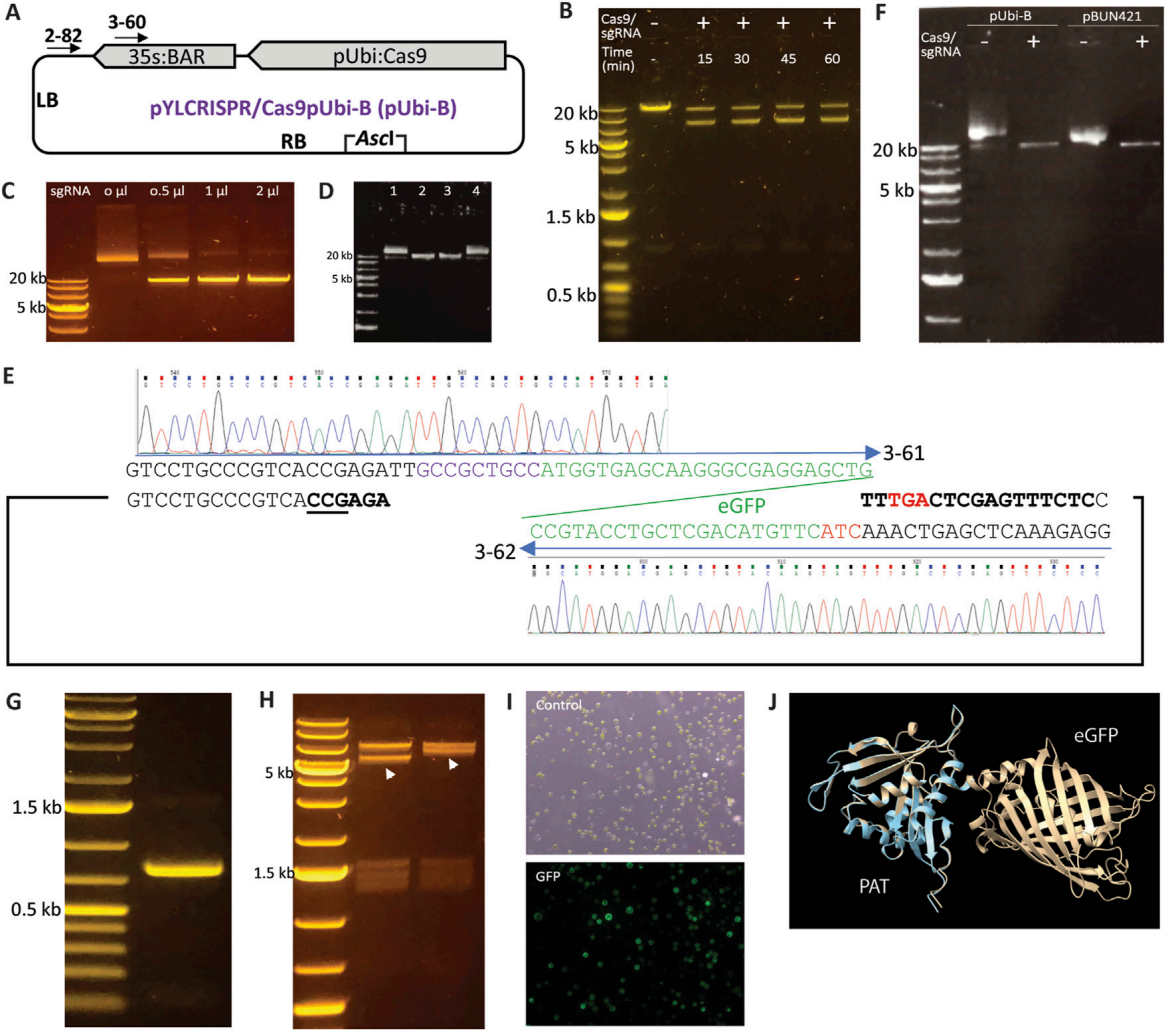
Modification of binary vectors using *Sp*Cas9 and sgRNA. **(A)** Diagram of the pYLCRISPR/Cas9pUbi-B plasmid (Ma et al., 2015). **(B)** Incomplete digestion of linearized pYLCRISPR/Cas9pUbi-B plasmid despite treatment with an overdose of the *Sp*Cas9 and sgRNA2-82 complex as well as extended incubation times (up to 60 min). One µg of plasmid substrate was treated with 0.5 µM each of *Sp*Cas9 and sgRNA2-82 in 80 µL reaction resulting in the molecular ratio between *Sp*Cas9 complex and plasmid more than 200-fold. **(C)** Digestion of a linearized plasmid with an sgRNA gradient; increasing the concentration of sgRNA (1 μg/μL) in the sgRNA/Cas9 RNP complex led to complete digestion of the substrate, indicating that sgRNA is the limiting factor for Cas9 digestion. **(D)** *Sp*Cas9 protects sgRNA from RNaseA digestion. Lane 1: The plasmid pYLCRISPR/Cas9pUbi-B was included in the reaction as a circular DNA control. Lane 2: *Sp*Cas9, sgRNA, and the plasmid were added simultaneously to the reaction to serve as a linear DNA control. Lane 3: *Sp*Cas9 and sgRNA were added first and incubated for 1 min before the addition of RNaseA and the plasmid substrate. Lane 4: RNaseA was added together with sgRNA and incubated for 1 min prior to adding the *Sp*Cas9 protein and plasmid. **(E)** Schematic representation of the fusion of the eGFP coding sequence to the BAR gene coding sequence within the pYLCRISPR/Cas9pUbi-B vector using the primers 3-61 and 3-62. **(F)** Linearization of the pUbi-B and pBUN421 vectors using the *Sp*Cas9 and sgRNA3-60 complex **(G)** Agarose gel displaying the amplicon of the eGFP coding sequence obtained from the modified pYLCRISPR/Cas9pUbi-B vector. **(H)** Restriction analysis of the pYLCRISPR/Cas9pUbi-B scaffold and clones containing the inserted eGFP coding sequence. The pYLCRISPR/Cas9pUbi-B vector and recombinant clones were digested with NotI, with the shifted fragment due to increased size by addition of GFP indicated by arrows. A comparable example of cloning GRF4-GIF1 into the pBUN421 vector is illustrated in Supplementary Figure S1. **(I)** Transformation of protoplasts using the modified pYLCRISPR/Cas9pUbi-Bvector containing eGFP resulted in GFP expression with a transformation efficiency of 51%. **(J)** The structural comparison between the PAT and the PAT-eGFP protein fusion is shown. The light blue ribbon represents PAT alone, while the brown ribbon represents the PAT-eGFP fusion.

As a single-turnover DNA endonuclease (Jinek et al., 2012), *Sp*Cas9 theoretically requires the RNP complex to have an equal or higher molecular count than the DNA substrate to achieve complete substrate cleavage. Anders and Jinek (2014) reported using a 25-fold excess of the *Sp*Cas9 and sgRNA complex relative to the DNA substrate. In our experiments, we used the pYLCRISPR/Cas9pUbi-B vector (Ma et al., 2015) as the test substrate and designed sgRNA2-82 targeting a non-specific region near the left border (Figure 1A; Table 1). To assess cleavage efficiency, we introduced the restriction enzyme *Asc*I into the reaction. Our initial attempts with the *Sp*Cas9-sgRNA duplex resulted in only partial digestion of the linearized plasmid (Figure 1B), even with extended incubation times and a significant overdose (approximately 200-fold) of the Cas9-sgRNA complex. Further testing with a gradient of sgRNA concentrations indicated that sgRNA was the limiting factor for DNA substrate cleavage (Figure 1C), likely due to imprecise sgRNA quantification or non-specific transcription by T7 RNA polymerase.

As an RNA molecule, sgRNA is susceptible to degradation by RNaseA. During plasmid or genomic DNA extraction, RNaseA is commonly used, and residual RNaseA in the DNA samples can degrade sgRNA added to the reaction. This degradation can inhibit *Sp*Cas9-mediated substrate cleavage, as residual RNaseA is often difficult to fully eliminate from the samples. Given the structure of the *Sp*Cas9 and sgRNA complex, we hypothesized that RNase A is unable to access the sgRNA once the *Sp*Cas9-sgRNA complex is formed. To investigate this, we conducted two digestions that differed only in the order of component addition. When *Sp*Cas9 protein was added prior to RNaseA, the DNA substrate could be efficiently cleaved. Conversely, when *Sp*Cas9 was added to sgRNA after RNaseA, the DNA substrate remained intact (Figure 1D). Therefore, *Sp*Cas9 can protect sgRNA from degradation by RNase, eliminating the need to remove residual RNase from DNA substrate samples.

Constructs employed in plant genetic engineering often pose challenges due to their size and limited unique restriction sites, particularly outside the multiple cloning sites. For instance, the pYLCRISPR/Cas9pUbi-B vector (Figure 1A), which, despite featuring a *Bsa*I site for cloning multiple sgRNAs, lacks unique restriction site(s) for efficiently adding reporter gene to the scaffold in a single cloning step. Recognizing the potential of *Sp*Cas9 and custom-prepared sgRNA for linearizing DNA constructs through induced double strand breaks, we hypothesized that this method could be effectively utilized to manipulate large constructs at specific loci. To validate this idea, we fused a PCR amplified fragment of eGFP coding sequence (Vickers et al., 2007; Figures 1E–H) to the coding sequence (CDS) of the bialaphos resistance (BAR) gene, encoding phosphinothricin acetyltransferase (PAT), in the pYLCRISPR/Cas9pUbi-B and pBUN421 vectors (Xing et al., 2014). The sgRNA3-60 was designed and utilized in combination with *Sp*Cas9 to linearize both the pYLCRISPR/Cas9pUbi-B and pBUN421 vectors at the end of the BAR gene’s CDS (Figure 1A), followed by insertion of the eGFP amplicon through ligation-based *in vitro* cloning. Restriction analysis of the recombined clone confirmed the successful cloning of eGFP into the pYLCRISPR/Cas9pUbi-B and pBUN421 vectors (Figure 1H). Using similar strategy, we also added GRF4-GIF1 cassette, a chimeric gene improving regeneration efficiency in monocot plants, into pBUN421 vector (Supplementary Figure S1). Sanger sequencing of the resulting clones revealed no mutations or mismatches at the boundary regions, and confirmed in-frame fusion of the coding sequences. Protoplasts transformed with the modified vector displayed eGFP expression, resulting in a transformation efficiency of 51% as determined by counting the fluorescent cells (Figure 1I). To determine whether the BAR-eGFP gene fusion would produce active phosphinothricin N-acetyltransferase (PAT) for transgene selection, we used AlphaFold3 (Abramson et al., 2024) to model the structure of the BAR-eGFP fusion protein. As shown in Figure 1J, generated using ChimeraX (Meng et al., 2023), the structure of PAT in both the BAR-coding sequence (CDS) alone and the BAR-eGFP fusion protein were highly similar, with no apparent changes in the active domains. Therefore, this binary vector containing the BAR-eGFP fusion gene is expected to produce functional PAT for transgene selection.

### 3.2 Detection of CRISPR edits using *Sp*Cas9 and custom-designed sgRNAs

**FIGURE 2:**
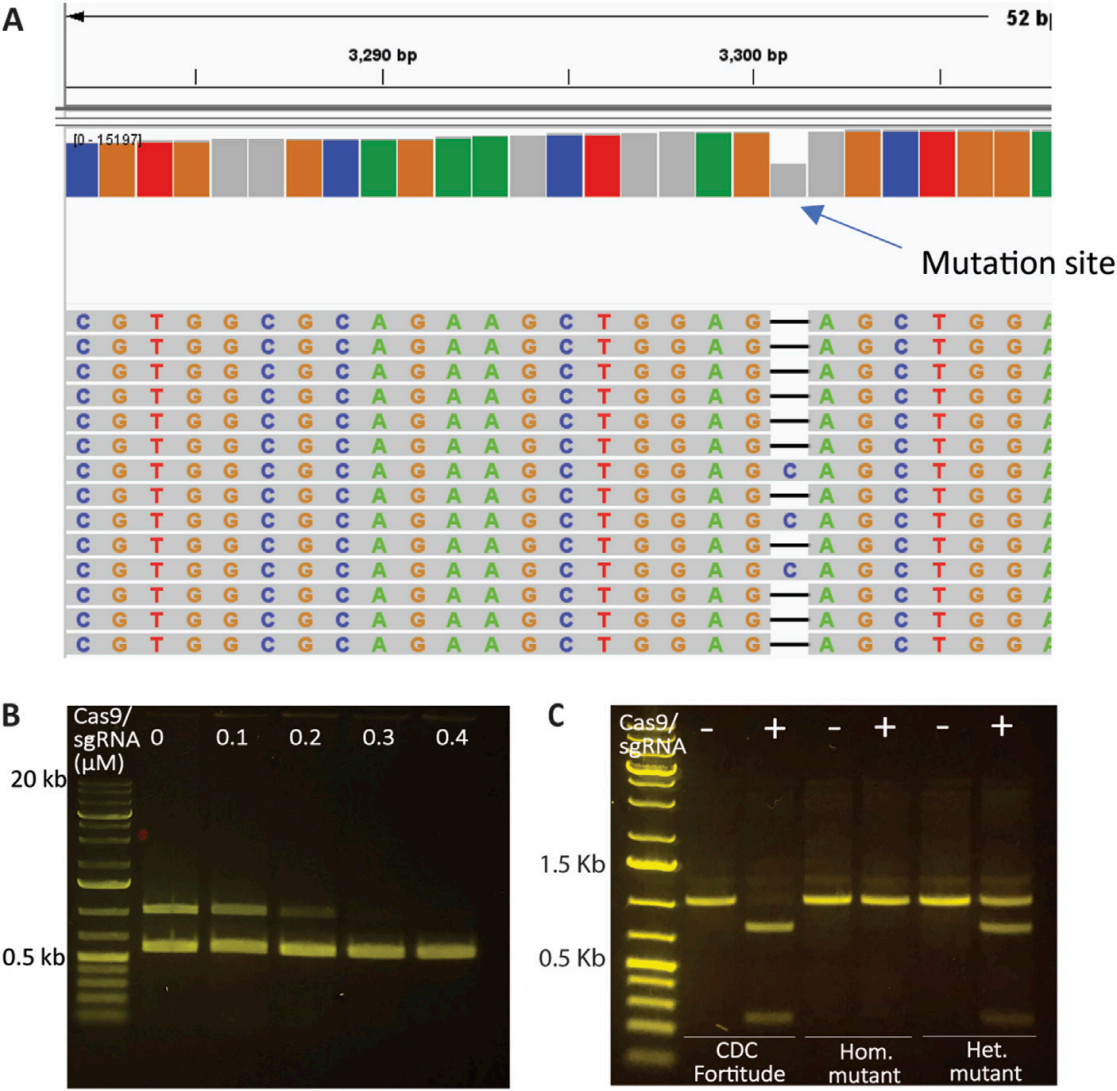
Assessment of CRISPR modifications using *Sp*Cas9 and sgRNAs. **(A)** Rht-B1 mutant in durum wheat carrying a 1 bp deletion in a heterozygous state, as validated by MiSeq. **(B)** Treatment of the genomic DNA of CDC Fortitude with a gradient of sgRNA4-2:*Sp*Cas9 RNP (targeting Rht-B1 only) complex followed by amplification of Rht-B1 and Q genes in the same reaction. Increasing the concentration of the *Sp*Cas9 and sgRNA complex led to complete elimination of the amplification of Rht-B1, while the amplification of Q gene fragments in the same reaction remained unaffected. **(C)** The utility of sgRNA4-2:*Sp*Cas9 RNP complex to detect mutations by treating PCR amplicons. The results indicate that the Rht-B1 amplicon from the wild type CDC Fortitude was fully digested at the target site, while the homozygous mutant (Hom.) exhibited resistance to digestion, and the heterozygous mutant (Het.) displayed partial digestion.

Next, we investigated the utility of *Sp*Cas9-based *in vitro* digestion, paired with PCR amplification, for detecting CRISPR/Cas9-induced edits. This approach resembles restriction resistance, where the *in vitro*-prepared sgRNA/Cas9 complex is expected to not bind to the edited substrate. This assumption was confirmed by identifying an Rht-B1 gene-edited mutant in durum wheat, which carried a 1 bp deletion in a heterozygous state, as validated by MiSeq (Figure 2A). The sgRNA4-2, designed to generate the mutation in Rht-B1, was used in combination with *Sp*Cas9 to treat genomic DNA samples from the wild type prior to PCR amplification. As shown in Figure 2B, increasing the concentration of the *Sp*Cas9 and sgRNA complex resulted in decreased amplification of Rht-B1, whereas the amplification of Q gene fragments in the same reaction remained unaffected. This result confirmed the effectiveness of the sgRNA4-2:*Sp*Cas9 RNP complex in successfully targeting and cleaving the Rht-B1 region in the wild type, and indicated that a minimum concentration of 0.3 µM RNP complex is required for complete DNA digestion. Furthermore, digestion of the Rht-B1 PCR amplicon resulted in complete digestion of the wild type, partial digestion of the heterozygous mutant, and no digestion of the homozygous mutant, thereby confirming the heterozygous status of the mutant (Figure 2C; Supplementary Figure S2). These results demonstrate the successful application of *Sp*Cas9-based *in vitro* digestion for detecting CRISPR/Cas9-induced gene edits.

In summary, the use of *Sp*Cas9 in conjunction with sgRNA for linearizing plasmids in homologous recombination-based cloning provides a robust method for construct manipulation. This method can also be adapted for fragment replacement by designing two sgRNA targets. For the mutant screening method, *Sp*Cas9 digestion of genomic DNA prior to PCR amplification of the target region can be employed, whereas performing digestion after amplification enables differentiation between biallelic and heterozygous mutants by analyzing agarose gel patterns in comparison to wild-type controls. This approach provides a straightforward and highly flexible method for modifying large constructs and screening CRISPR-induced edits.

## Data Availability

The datasets presented in this study can be found in online repositories. The names of the repository/repositories and accession number(s) can be found in the article/Supplementary Material.
