# Supplementary material for: Cas9 endonuclease: a molecular tool for in vitro cloning and CRISPR edit detection
Source: Front Genome Ed. 2025 Apr 1;7:1565297. doi: 10.3389/fgeed.2025.1565297 (PMC11996781; doi:10.3389/fgeed.2025.1565297)
Supplement: Supplementary file 1 [file Supplementaryfile1.docx]

Supplementary Material

# Supplementary Note

**Protein sequences of BAR and BAR-eGFP products**

***BAR gene product (PAT):***

MSPERRPADIRRATEADMPAVCTIVNHYIETSTVNFRTEPQEPQEWTDDLVRLRERYPWLVAEVDGEVAGIAYAGPWKARNAYDWTAESTVYVSPRHQRTGLGSTLYTHLLKSLEAQGFKSVVAVIGLPNDPSVRMHEALGYAPRGMLRAAGFKHGNWHDVGFWQLDFSLPVPPRPVLPVTEI

***BAR-eGFP fusion gene product:***

MSPERRPADIRRATEADMPAVCTIVNHYIETSTVNFRTEPQEPQEWTDDLVRLRERYPWLVAEVDGEVAGIAYAGPWKARNAYDWTAESTVYVSPRHQRTGLGSTLYTHLLKSLEAQGFKSVVAVIGLPNDPSVRMHEALGYAPRGMLRAAGFKHGNWHDVGFWQLDFSLPVPPRPVLPVTEIAAAMVSKGEELFTGVVPILVELDGDVNGHKFSVSGEGEGDATYGKLTLKFICTTGKLPVPWPTLVTTFTYGVQCFSRYPDHMKQHDFFKSAMPEGYVQERTIFFKDDGNYKTRAEVKFEGDTLVNRIELKGIDFKEDGNILGHKLEYNYNSHNVYIMADKQKNGIKVNFKIRHNIEDGSVQLADHYQQNTPIGDGPVLLPDNHYLSTQSALSKDPNEKRDHMVLLEFVTAAGITHGMDELYK

# Supplementary Figures and Tables

## Supplementary Figures

**Supplementary Figure S1.** **Cas9-based in vitro cloning of GRF4-GIF1 and eGFP into the pBUN421 Vector**

A) Diagram showing the fusion of the GRF4-GIF1 with the BAR gene coding sequence in the pBUN421 vector using primers 3-23 and 3-24.

B) Restriction analysis of the pBUN421 scaffold and clones containing the GRF4-GIF1 cassette. The pBUN421 vector and recombinant clones were digested with EcoRV, and the shifted fragment indicating the increased size due to the GRF4-GIF1 insertion is marked with arrows.

C) Restriction analysis of the pBUN421 scaffold and clones with the eGFP coding sequence inserted. The pBUN421 vector and recombinant clones were digested with EcoRV, and the shifted fragment reflecting the increased size from the eGFP insertion is indicated by arrows.


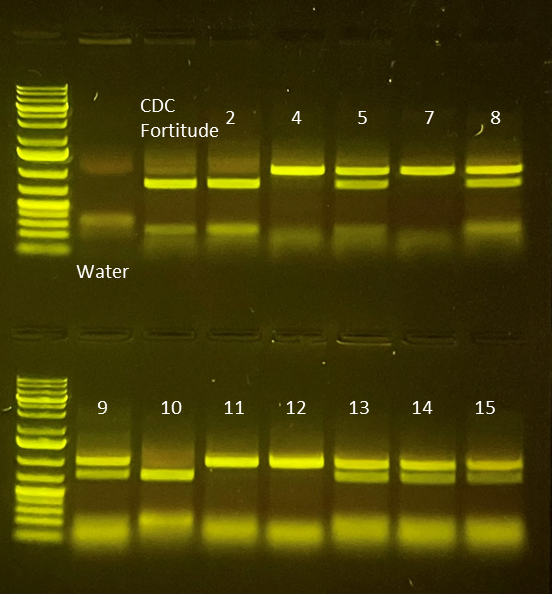


**Supplementary Figure S2. Progenies from the Rht-B1 mutant in durum wheat carrying a 1 bp deletion in homozygous, heterozygous, and wild type state.** Among the progenies, samples 2 and 10 exhibited an identical digestion pattern to the wild-type CDC Fortitude. samples 4, 7, 11, and 12 were completely resistant to digestion and hence homozygous, while samples 5, 8, 9, 13, 14, and 15 were heterozygous.
